# ﻿Two new species of crab spiders from Xiaolong Mountains in Gansu Province, China (Araneae, Thomisidae)

**DOI:** 10.3897/zookeys.1160.103644

**Published:** 2023-05-03

**Authors:** Rui Zhang, Feng Zhang

**Affiliations:** 1 Key Laboratory of Zoological Systematics and Application of Hebei Province, Institute of Life Science and Green Development, College of Life Sciences, Hebei University, Baoding, Hebei 071002, China Hebei University Baoding China

**Keywords:** *Ebelingia*, *Lysiteles*, new species, taxonomy

## Abstract

Two new species of crab spider are described from the Xiaolong Mountains in Gansu Province, China: *Ebelingiaspirala***sp. nov.** (♂♀) and *Lysiteleslongensis***sp. nov.** (♂♀). Detailed morphological characters, a distribution map, photographs, and illustrations of the habitus and copulatory organs are given for each species.

## ﻿Introduction

As the seventh largest family of spiders worldwide, Thomisidae Sundevall, 1833 currently contains 171 genera and 2710 species from all over the world (WSC 2023). It has undergone regional revisions in Canada (Dondale and Redner 1978), Japan (Ono 2009), and China (Song et al. 1999; Tang et al. 2007, 2008; Tang and Li 2010a, b). Although crab spiders have been revised, species reassigned, and unknown sexes described in recent decades, there are still many species needing in-depth study (Liu et al. 2022).

The genus *Ebelingia* Lehtinen, 2004 and *Lysiteles* Simon, 1895 are mainly distributed in eastern and southern Asia. Currently, only three species of *Ebelingia* are known (WSC 2023): *E.forcipata* Song & Zhu, 1993, *E.hubeiensis* Song & Zhao, 1994, and *E.kumadai* Ono, 1985. All three species are distributed in China. *E.forcipata* and *E.hubeiensis* are endemic to China, mainly distributed in Fujian, Hubei, and Jiangxi provinces. There are no reports of this genus in Gansu Province. There are 63 *Lysiteles* species worldwide. The Chinese *Lysiteles* fauna is extraordinarily rich with 45 species (WSC 2023). More than half of the species are distributed in southern China, such as Yunnan, Guizhou, and Hubei Provinces, and Hainan Island. Only three species were reported in Gansu Province.

To enrich the diversity of *Ebelingia* and *Lysiteles* in Gansu Province, a survey from Xiaolong Mountains was carried out by colleagues of Hebei University. After a careful examination of thomisid materials, two new species, *Ebelingiaspirala* sp. nov. and *Lysiteleslongensis* sp. nov., were recognized. Illustrations of diagnostic structures and a distribution map are presented.

## ﻿Materials and methods

All specimens are preserved in 95% ethanol. Specimens were examined and measured under a Leica M205A stereomicroscope. Photographs were taken using an Olympus BX51 microscope equipped with a Kuy Nice CCD and were imported into Helicon Focus v. 7 for image stacking. Final figures were retouched using Adobe Photoshop 2020. Eye sizes were measured as the maximum diameter in dorsal view. Leg measurements are shown as total length (femur, patella, tibia, metatarsus, and tarsus). All measurements are given in millimetres. The holotypes of the new species are deposited in the Museum of Hebei University (**MHBU**), Baoding, China. The paratypes are in the Museum of Baoding University.

Abbreviations used: **AME**, anterior median eyes; **ALE**, anterior lateral eyes; **AME–ALE**, distance between AME and ALE; **AME–AME**, distance between AMEs; **PME**, posterior median eyes; **PME–PLE**, distance between PME and PLE; **PME–PME**, distance between PMEs; **PLE**, posterior lateral eyes.

## ﻿Taxonomy

**Family Thomisidae Sundevall, 1833**

### ﻿Genus Ebelingia

Taxon classificationAnimaliaAraneaeThomisidae

Lehtinen, 2004

76BED021-9288-56CF-B1D6-51C8C1AA4652

#### Type species.

*Misumenopskumadai* Ono, 1985 from Japan.

#### Diagnosis.

See Lehtinen (2004).

#### Comments.

This genus includes only three species, all of which are distributed in East Asia. Among them, *E.forcipata* and *E.hubeiensis* are endemic to China and recorded from Fujian, Hubei, and Jiangxi provinces. No species were recorded from Gansu Province.

#### Distribution.

China, Japan, Korea, Russia (Far East).

### ﻿ Ebelingia spirala sp. nov.

Taxon classificationAnimaliaAraneaeThomisidae

CAC7CAFE-DE20-5082-81E6-AFE6A3CDFD30

https://zoobank.org/95C67764-CC97-4FE4-87BE-29FC005D40C4

Figs 1–4 , 5, 6 , 7–8

#### Type material.

***Holotype***: ♂, China: Gansu Province, Maiji district, Liqiao town, Baihua Forest Farm, 34°19.93'N, 106°23.18'E, 1844 m, 23 May 2021, Rui Zhang leg. ***Paratypes***: 1♂3♀, with same data as holotype; 2♀, Niangniangba town, Baiyin village, 34°17.2'N, 105°55.97'E, 1524 m, 31 May 2021, Rui Zhang leg; 1♀, Hui County, Jialing town, Xiaolongshan National Nature Reserve, 33°40.52'N, 106°18.67'E, 1647 m, 7 June 2021, Zhaoyi Li leg.; 1♂, Qingshui County, Shanmen Town, Shanmen Village, 34°41.4'N, 106°21.72'E, 1630 m, 24 June 2022, Zhaoyi Li leg.

#### Etymology.

The specific name is derived from the Latin “*spira*” (meaning “a coil”), referring to the shape of RTA in ventral view, adjective.

#### Diagnosis.

Male of this new species resembles those of *E.forcipata* Song & Zhu, 1993 (see Liu et al. 2022: 51, figs 4A–E, 5A–F) and *E.hubeiensis* Song & Zhao, 1994 (see Song and Zhao 1994: 115, fig. 4E, F) in having short embolus, flat tegulum, and a bifurcated RTA, but can be distinguished by the following combination of characters: (1) RTA about half the length of tibia (vs almost as long as tibia); (2) the presence of spiral thread on dorsal branch of RTA (vs smooth RTA). Female of *E.spirala* sp. nov. is similar to that of *E.hubeiensis* in having central concavity on anterior hood but can be distinguished by the L-shaped, long spermathecae (vs same length and width in *E.hubeiensis*).

**Figures 1–4.**
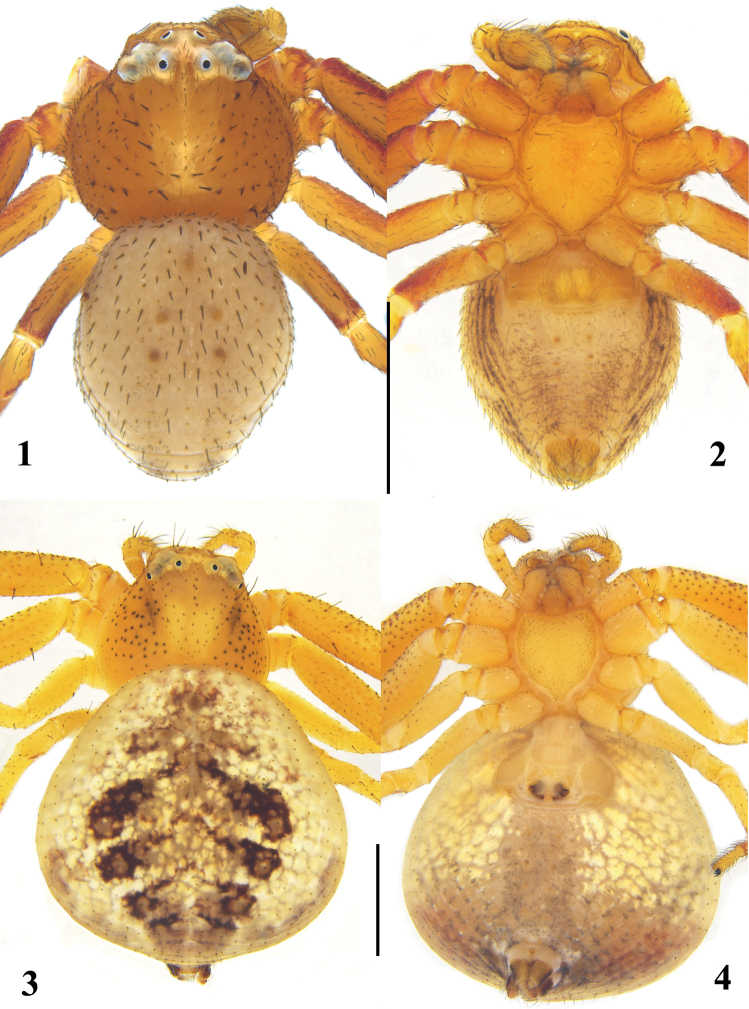
*Ebelingiaspirala* sp. nov. **1, 2** male habitus (**1** dorsal view **2** ventral view) **3, 4** female habitus (**3** dorsal **4** ventral). Scale bars: 1 mm.

#### Description.

**Male (*holotype*).** Habitus as in Figs 1, 2. Total length 2.88. Carapace 1.27 long, 1.21 wide, opisthosoma 1.60 long, 1.22 wide, the whole dorsum of body with dense setae. Carapace chestnut-coloured, medially with yellowish band. Ocular area white. Eye sizes and interdistances: AME 0.06, ALE 0.09, PME 0.05, PLE 0.08, AME–AME 0.14, AME–ALE 0.15, PME–PME 0.20, PME–PLE 0.22, AME–PME 0.15, ALE–PLE 0.16. MOA 0.18 long, front width 0.27, back width 0.50. Sternum slightly longer than wide. Chelicerae, endites, and labium yellow. Femora and patellae of legs I–II and legs III–IV reddish brown, other segments of legs I–II dark brown. Leg measurements: I 5.73 (1.59, 0.62, 1.52, 1.43, 0.57); II 5.63 (1.68, 0.56, 1.32, 1.38, 0.69); III 2.34 (0.74, 0.30, 0.48, 0.49, 0.32); IV 2.52 (0.66, 0.36, 0.54, 0.62, 0.34). Leg spination: I Fe: p2; II Fe: d2; III Fe: d2; Ti: d1; IV: Pa: d1; Ti: d2. Opisthosoma dorsum yellowish, with cardiac pattern, posterior with irregular stripes; venter yellow, with black stripes.

**Figures 5, 6.**
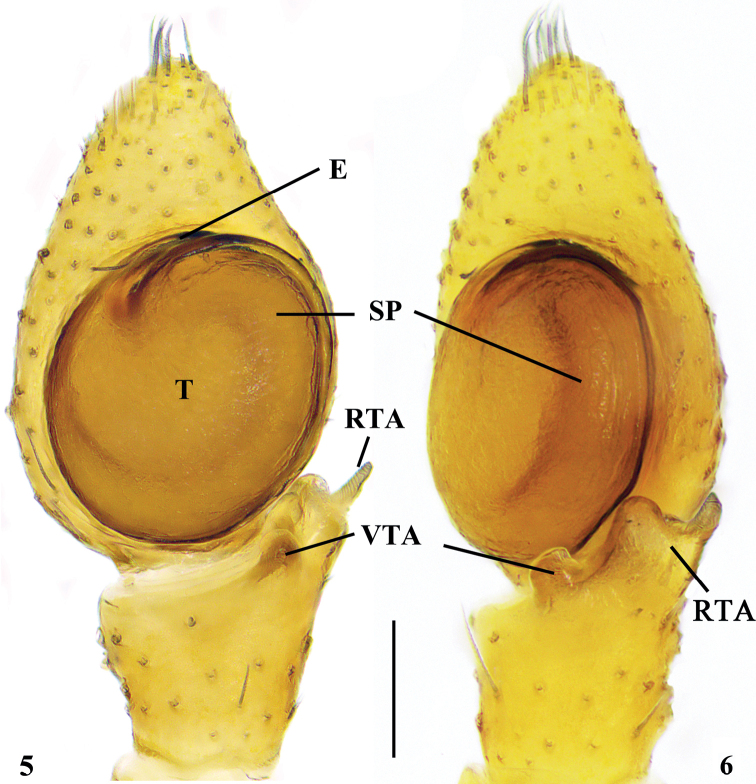
*Ebelingiaspirala* sp. nov. **5, 6** left male palp (**5** ventral view **6** retrolateral view). E = embolus; RTA = retrolateral tibial apophysis; SP = spermophore; T = tegulum; VTA = ventral tibial apophysis. Scale bar: 0.1 mm.

Palp (Figs 5, 6). Tibia with two apophyses, short ventral and bifurcated retrolateral: ventral part blunt and longer than ventral tibial apophysis in retrolateral view, dorsal one with spirals. Ventral tibial apophysis short, with blunt apex. Cymbium 1.25× longer than wide. Tegulum oval 1.25× longer than wide, tegular ridge at 11 o’clock position. Spermophore wide, encircling almost whole tegulum. Embolus short, originating from ~11 o’clock position and terminating at 1 o’clock position.

**Female.** Habitus as in Figs 3, 4. Total length 4.38. Prosoma 1.53 long, 1.57 wide. Opisthosoma 2.85 long, 2.88 wide. Carapace chestnut-coloured, medially with yellowish band, laterally with black spots. Eye sizes and interdistances: AME 0.06, ALE 0.09, PME 0.05, PLE 0.10, AME–AME 0.19, AME–ALE 0.17, PME–PME 0.29, PME–PLE 0.26, AME–PME 0.20, ALE–PLE 0.17. MOA 0.27 long, front width 0.31, back width 0.39. Chelicerae, sternum, and labium yellow. Endites and legs chestnut-coloured. Venter of leg I and II with numerous reddish-brown spots. Leg measurements: I 5.58 (1.77, 0.74, 1.30, 1.12, 0.65); II 5.14 (1.24, 0.75, 1.37, 1.09, 0.69); III 2.60 (0.78, 0.43, 0.70, 0.39, 0.30); IV 2.86 (0.89, 0.45, 0.58, 0.53, 0.41). Leg spination: I Fe: d1, p4; Pa: d2; Ti: v3; Mt: p4, r4; II Fe: d1; Pa: d1; Ti: p2, r3; Mt: d3, p5, r5; III Fe: d1; Pa: d1; Ti: d3, v4; IV: Fe: d2; Pa: d2; Ti: d2, v2. Opisthosomal dorsum yellow, with white spots at the sides and brown symmetrical patches in the middle; venter with a few white spots at the sides.

**Figures 7, 8.**
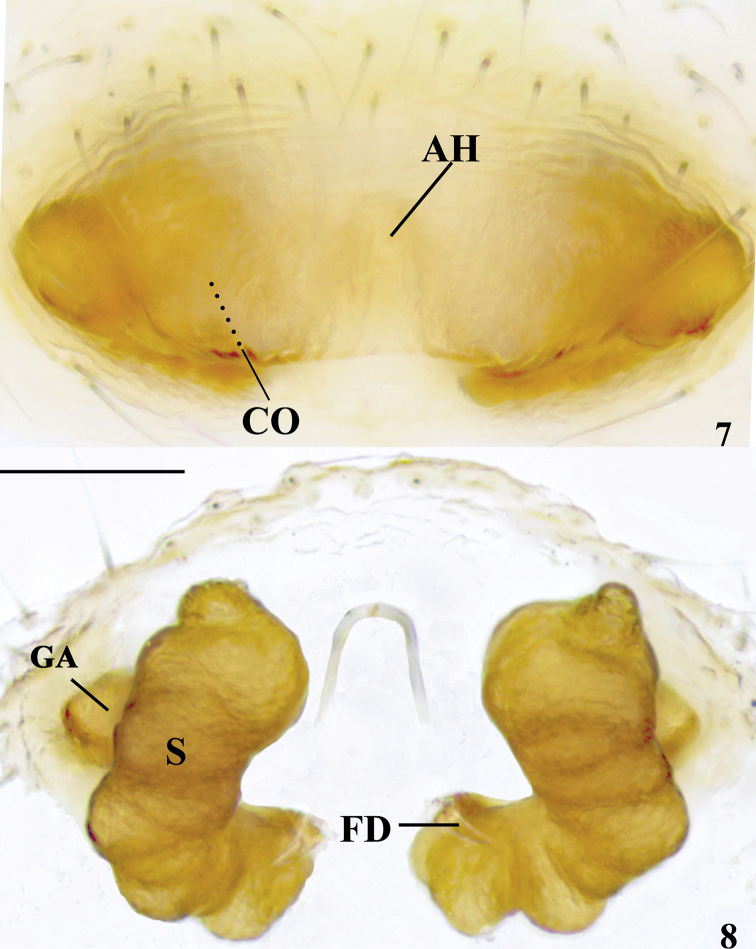
*Ebelingiaspirala* sp. nov. **7, 8** epigyne/vulva (**7** ventral view **8** dorsal view). AH = anterior hood; CO = copulatory opening; FD = fertilization duct; GA = glandular appendage; S = spermatheca. Scale bar: 0.1 mm.

Epigyne (Figs 7, 8). Epigyne almost 2× wider than long, with a deep Ո-shaped anterior hood, about 2× longer than wide. Copulatory openings (Fig. 7) located at posterolateral part of anterior hood. Spermathecae L-shaped, separated by more than width of anterior hood. Fertilization ducts short.

#### Distribution.

Known only from the type locality in Gansu Province, China (Fig. 17).

### ﻿Genus Lysiteles

Taxon classificationAnimaliaAraneaeThomisidae

Simon, 1895

B04CBFC0-CC5A-5F93-AA0D-0DDA2ACA999C

#### Type species.

*Lysitelescatulus* Simon, 1895 from Tamil Nadu, India.

#### Diagnosis.

See Tang et al. (2007) and Tang and Li (2010a, b).

#### Comments.

This genus includes 63 species mainly distributed in eastern Asia. It has never been revised in full, although new species have been described now and then in various papers. Most of the 44 *Lysiteles* species have been recorded from China, and three species have been recorded from Gansu Province.

#### Distribution.

Bhutan, China, India, Japan, Korea, Nepal, Pakistan, Philippines, Russia, Vietnam.

### ﻿ Lysiteles longensis sp. nov.

Taxon classificationAnimaliaAraneaeThomisidae

261EBE43-210B-5732-B736-CA7E0F662DD1

https://zoobank.org/873582B1-1E1F-48AC-91F7-76FE3C658BE6

Figs 9–12 , 13, 14 , 15–16

#### Type material.

***Holotype***: ♂, China: Gansu Province, Qingshui County, Shanmen town, Shanmen village, 34°40.72'N, 106°23.98'E, 1735 m, 22 May 2021, Rui Zhang leg. ***Paratypes***: 2♂6♀, with same data as holotype; 2♂9♀, Dangchuan town, 34°19.73'N, 105°15.77'E, 1711 m, 24 May 2021, Zhaoyi Li leg.; 1♂3♀, Qingshui County, Shanmen town, Shanmen village, 34°42.32'N, 106°25.1'E, 1635 m, 3 August 2021, Rui Zhang leg.; 3♂6♀, Qingshui County, Shanmen town, Daji village, 34°37.45'N, 106°20.25'E, 1784 m, 23 June 2022, Xinyuan Bai leg.

#### Etymology.

The specific name refers to the type locality. “Long” is a short name for Gansu, adjective.

#### Diagnosis.

Male *L.longensis* sp. nov. is similar to that of *L.silvanus* Ono, 1980 (see Ono 1980: 212, figs 28–30) in having a long RTA and twisted embolus, but it differs by the following combination of characters: (1) tegulum large and reniform, ca 3/4 of cymbium cavity (vs small, semicircular, and ca 1/2 of cymbium cavity); (2) the lowest point of embolus above the tegulum (vs the lowest point at 1/2 of the tegulum); (3) RTA straight, pointing dorsally (vs RTA flexed, pointing ventrally). Female is similar to that of *L.silvanus* (see Ono 1980: 212, figs 25–27) in having a broad atrium with a sclerotized, transversally extending plate and widely separated copulatory openings, but it can be easily distinguished by the short, thick, and spherical copulatory duct (vs slender and strongly twisted).

#### Description.

**Figures 9–12.**
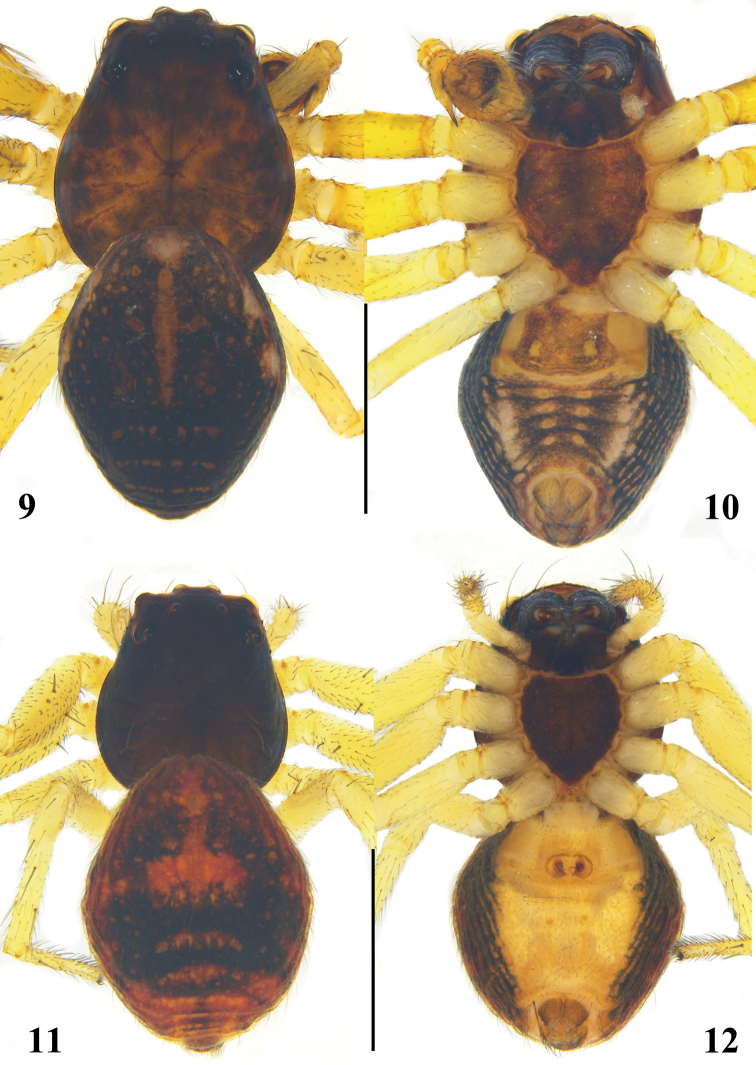
*Lysiteleslongensis* sp. nov. **9, 10** male habitus (**9** dorsal view **10** ventral view) **11, 12** female habitus (**11** dorsal view **12** ventral view). Scale bars: 1 mm.

**Male (*holotype*).** Habitus as in Figs 9, 10. Total length 2.89. Carapace 1.31 long, 1.19 wide. Opisthosoma 1.58 long, 1.19 wide. Carapace reddish brown. Eye sizes and interdistances: AME 0.06, ALE 0.11, PME 0.04, PLE 0.08, AME–AME 0.14, AME–ALE 0.13, PME–PME 0.25, PME–PLE 0.24, AME–PME 0.25, ALE–PLE 0.23. MOA 0.24 long, front width 0.26, back width 0.33. Chelicerae, labium and maxillae blackish brown. Legs yellowish and spinous. Leg measurements: I 4.83 (1.37, 0.46, 1.18, 1.06, 0.76); II 5.06 (1.41, 0.55, 1.28, 1.04, 0.78); III 2.88 (0.86, 0.40, 0.79, 0.45, 0.38); IV 2.93 (0.84, 0.39, 0.82, 0.49, 0.39). Leg spination: I Fe: d3, p3; Ti: d3, p1, r1, v4; Mt: p3, r3, v1; II Fe: d3, p2; Pa: d2, p1, r1; Ti: d1, p3, r2, v3; Mt: p3, r3, v4; III Fe: d3; Pa: d1, r1; Ti: d1, p2, r1; Mt: p2, r1; IV: Fe: d4; Pa: d1, r1; Ti: d2, p2, r2; Mt: p1, r1. Opisthosoma dorsum blackish brown, anterior with longitudinal reddish-brown stripe, posterior part with 3 transverse, reddish-brown stripes, and lateral with many small, scattered, brown spots; venter with 4 pairs of longitudinal yellow spots in the middle; spinnerets brown.

**Figures 13, 14.**
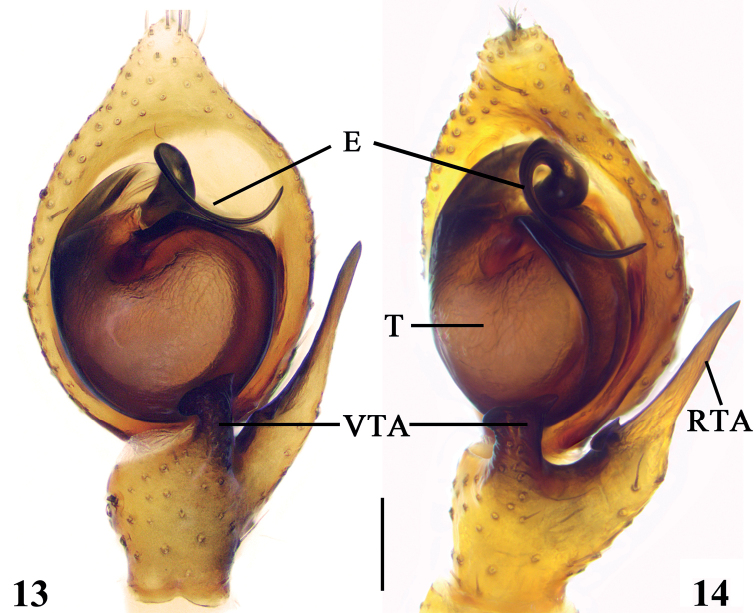
*Lysiteleslongensis* sp. nov., left male palp (**13** ventral view **14** retrolateral view). E = embolus; RTA = retrolateral tibial apophysis; T = tegulum; VTA = ventral tibial apophysis. Scale bar: 0.1 mm.

Palp (Figs 13, 14). Retrolateral tibial apophysis longer than tibia, with small, basal protuberance (Fig. 14), apically pointed; ventral tibia apophysis digitiform, 1.5× longer than wide, short and broad, apically curved; extending along tegular margin. Embolus with thick base and a strong, dorsally bent apical end. Tip lopped and in counterclockwise direction, base at about 10 o’clock, tip at about 2 o’clock; tegulum as long as wide.

**Female.** Habitus as in Figs 11, 12. Total length 3.27. Prosoma 1.38 long, 1.17 wide. Opisthosoma 1.89 long, 1.60 wide. Carapace blackish brown. Other characteristics as those of males. Eye sizes and interdistances: AME 0.06, ALE 0.09, PME 0.05, PLE 0.10, AME–AME 0.19, AME–ALE 0.17, PME–PME 0.29, PME–PLE 0.26, AME–PME 0.20, ALE–PLE 0.17. MOA 0.27 long, front width 0.31, back width 0.39. Sternum, chelicerae, labium, and maxillae blackish brown. Legs yellowish and spinous. Leg measurements: I 4.26 (1.32, 0.38, 0.99, 0.96, 0.61); II 4.20 (1.31, 0.41, 1.00, 0.91, 0.57); III 2.49 (0.58, 0.33, 0.68, 0.50, 0.40); IV 2.71 (0.65, 0.40, 0.68, 0.55, 0.43). Leg spination: I Fe: d1, p3; Pa: d1; Ti: d3, p2, r3, v1; Mt: p4, r4; II Fe: d2; Ti: d2, p2, r2, v1; Mt: p3, r3, v2; III Fe: d2; Pa: d1; Ti: d2, p2, r2; Mt: p2, r2, v1; IV: Fe: d1; Pa: d1; Ti: d1, p2, r1; Mt: p1, r1. Opisthosoma dorsum with symmetrical, longitudinal, reddish-brown stripes and lateral with scattered patches. Venter yellow in the middle and black at the sides.

**Figures 15, 16.**
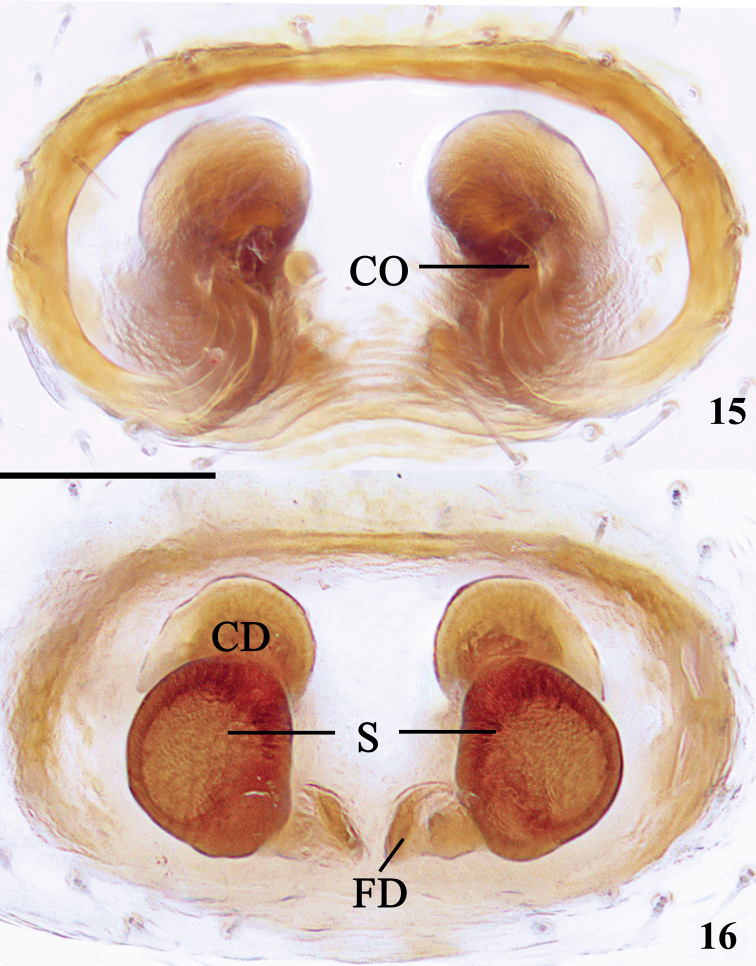
*Lysiteleslongensis* sp. nov., epigyne/vulva (**15** ventral view **16** dorsal view). CD = copulatory duct; CO = copulatory opening; FD = fertilization duct; S = spermatheca. Scale bar: 0.1 mm.

Epigyne (Figs 15, 16). Epigyne ca 1.7× wider than long. Atrium large, sclerotized plate oval, copulatory openings originate medially; sclerotic fold well developed. Copulatory ducts thick, as wide as spermathecae; spermathecae suboval, 1.4× wider than long, spaced by about 0.5 diameters of the vulva.

#### Distribution.

**Figure 17.**
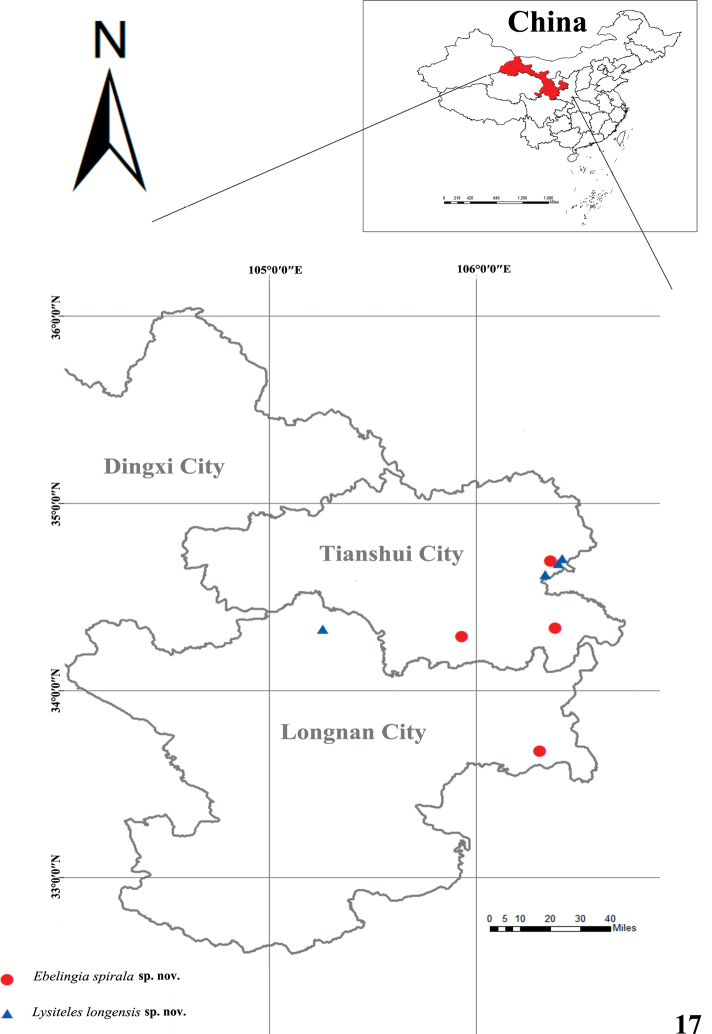
Records of the new species from the Xiaolong Mountains in Gansu Province, China.

Known only from the type locality in Gansu Province, China (Fig. 17).

## Supplementary Material

XML Treatment for Ebelingia

XML Treatment for Ebelingia spirala

XML Treatment for Lysiteles

XML Treatment for Lysiteles longensis
